# Esophageal granular cell tumor colliding with intramucosal adenocarcinoma: a case report

**DOI:** 10.4076/1757-1626-2-8093

**Published:** 2009-07-29

**Authors:** Fuad Alkhoury, Jeremiah T Martin, Paul Fiedler, Philip E Jaffe

**Affiliations:** Department of Surgery, Hospital of St. Raphael1450 Chapel Street, New Haven, CT 06511USA

## Abstract

We report a case of a granular cell tumor colliding with intramucosal adenocarcinoma of the esophagus. A 58-year-old white was found to have a 5 mm nodule in the distal esophagus detected by upper gastrointestinal endoscopy performed as part of the workup of long standing reflux. Endoscopic biopsies revealed intramucosal adenocarcinoma arising in the setting of Barrett’s esophagus. The adenocarcinoma infiltrated a granular cell tumor also present at the nodular site. Endoscopic mucosal resection using Duette band ligation and hot snare electrocautery was performed. Margins were negative for both tumors, and endoscopic surveillance for recurrence is planned.

## Introduction

Granular cell tumors (GCTs) are relatively uncommon, usually benign neoplasms, which may arise anywhere in the body [1]. About 300 cases of esophageal GCTs have been reported [2] and their possible association with malignant tumors in various organs has been suggested [3]. However, we were unable to find any report of GCT associated with another malignancy in the same region of the esophagus. To our knowledge, this is the first published report of a GCT colliding with adenocarcinoma of the esophagus.

## Case presentation

**Figure 1.**
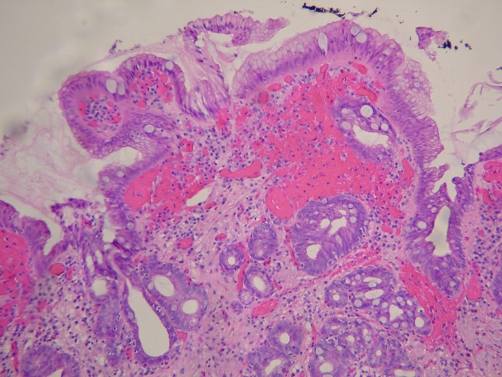
Barrett’s esophagus (H&E stain, low power).

**Figure 2.**
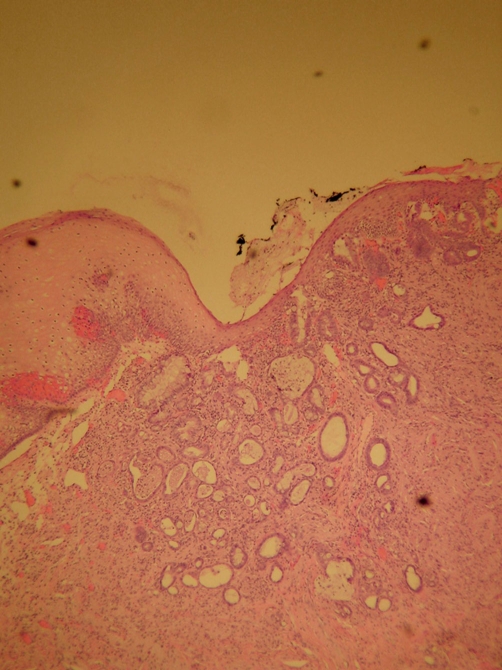
Intramucosal adenocarcinoma colliding with granular cell tumor (H&E stain, low power).

**Figure 3.**
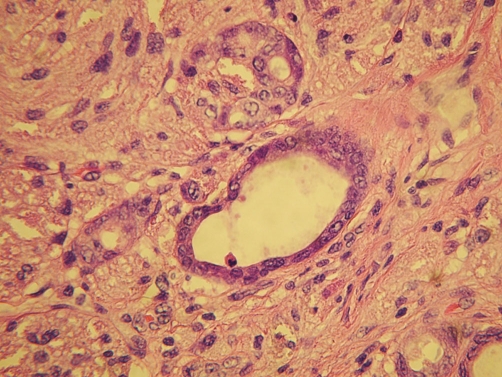
Intramucosal adenocarcinoma colliding with granular cell tumor (H&E stain, high power).

**Figure 4.**
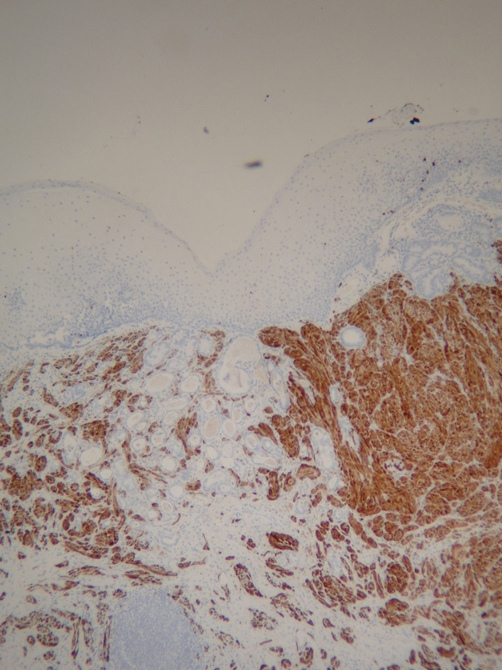
Intramucosal adenocarcinoma (pale counter-stained glands near center) colliding with S100+ granular cell tumor (S100 immunostain, low power).

**Figure 5.**
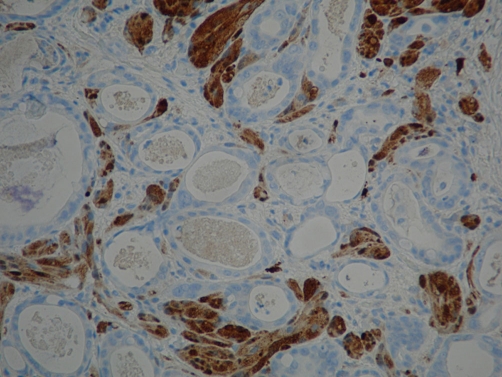
Intramucosal adenocarcinoma (pale counter-stained glands) colliding with S100+ granular cell tumor (S100 immunostain, high power).

**Figure 6.**
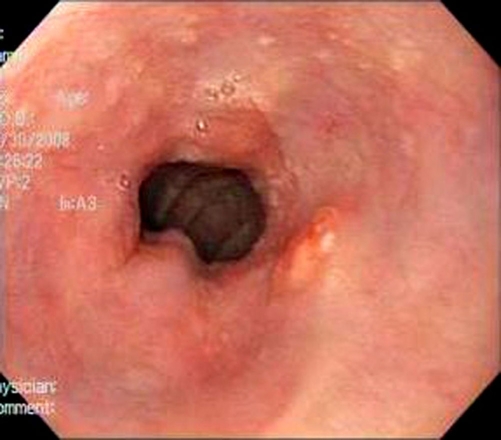
Slightly raised nodule is seen just proximal to the esophagogastric junction.

**Figure 7.**
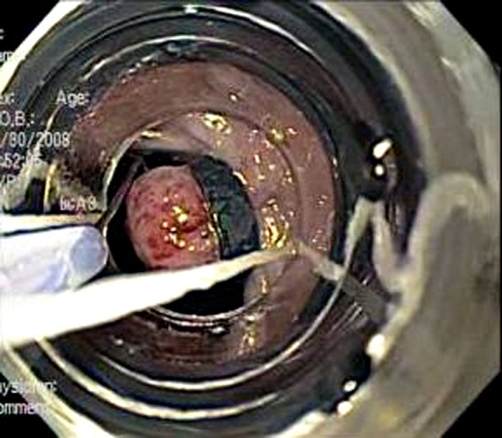
Pseudopolyp created following endoscopic suction and band application visualized through Cook Endoscopy Duette® Multi-Band Mucosectomy Device.

**Figure 8.**
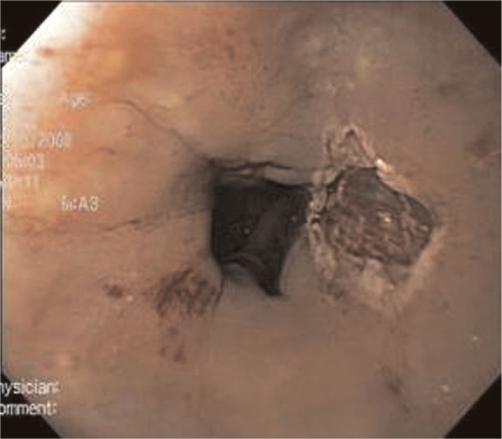
Mucosal defect following snare electrocautery resection.

A 58-year-old white man was found to have a 5 mm nodule in the distal esophagus 1 cm proximal to the Z-line when he underwent upper endoscopy during the workup of long standing reflux esophagitis. Biopsies from the lesion showed a granular cell tumor and Barrett’s esophagus without dysplasia. On follow up endoscopy with biopsies, adenocarcinoma was diagnosed arising in the setting of Barrett’s esophagus and colliding with a granular cell tumor (Figures 1-5). The patient was referred to our institution, the Hospital of St. Raphael, to consider endoscopic resection of the lesion. He was eager to avoid esophagectomy given his history of prior cerebrovascular accident, peripheral vascular disease, and his own reluctance to undergo a major resection. Staging CT scan revealed no evidence of local invasion, regional lymphadenopathy or metastatic disease. Endoscopic ultrasonography revealed no evidence of invasion beyond the mucosal wall layer. Therefore he was felt to be a suitable candidate for endoscopic mucosal resection (EMR) which was performed using a Cook Endoscopy Duette® Multi-Band Mucosectomy Device. (Figures 6-8). The patient tolerated the procedure well and was able to return home following the procedure.

On final pathology, the adenocarcinoma was confined to the mucosa, and margins were negative for both adenocarcinoma and granular cell tumor. Repeat endoscopy 8 weeks following mucosal resection revealed no evidence of a visible lesion at the site of the resected nodule. Biopsies from the esophagogastric junction revealed intestinal metaplasia without dysplasia. He will continue his outpatient surveillance at the referring institution, with our recommendation being endoscopy at 6 month intervals for 2 years, with interval increase to 2 year surveillance if no dysplasia is detected during the initial screening period.

## Discussion

Granular cell tumors are uncommon tumors, and only 1%-8% of them originate in the gastrointestinal tract. Esophageal GCTs account for about one third of all gastrointestinal GCTs [4]. In a review by Orlowska et al. [5], most (65%) esophageal GCTs were found in the distal esophagus, and the remaining 20% and 15% were found in the middle and proximal esophagus, respectively. Most were solitary, but up to 11% were multifocal.

Esophgeal GCTs are usually discovered incidentally during upper gastrointestinal endoscopy performed for other reasons. If symptoms are present; they are unrelated to tumor development in 50%-90% of patients [4,6]. Endoscopic ultrasonography may provide important diagnostic information including tumor size, layer of origin, and extension. The endoscopic appearance is often that of a small, usually less than 20 mm, yellowish-white, firm, sessile submucosal lesion covered by intact overlying mucosa, as in our case. When biopsies are taken too superficially, allowing only evaluation of the epithelium, the overlying hyperplastic squamous epithelium showing so-called “pseudoepitheliomatous hyperplasia” may lead to an erroneous diagnosis of well differentiated squamous cell carcinoma. Inappropriate esophagectomy may ensue [5,7,8].

There is an interesting but unexplained association between GCTs and other malignant neoplasms. Strong et al. reported that the incidence of this association was 9% [3]. The coexistence of GCT and a true malignant neoplasm in the same organ was described as an association of GCT with squamous cell carcinoma of the esophagus [8-10], tongue,[11] and larynx; [12] and GCT with adenocarcinoma in the bronchi [13], stomach [14], and breast [15]. Two of these cancers arose from the epithelium overlying the GCT [12,14]; however; we could not find any prior report of a GCT colliding with adenocarcinoma in the esophagus. On the other hand there are reports of carcinoma over other benign submucosal tumors (SMTs) of the esophagus, such as leiomyoma and lipoma with protrusion. Some investigators speculate that the pathogenesis of the overlying carcinoma may stem from chronic irritation of the esophageal mucosa, caused by intraluminal protrusion of the SMT [16,17].

The optimal treatment for GCTs remains controversial, however, the current treatment options are as follows: a conservative approach with regular endoscopic follow-up for tumors <10 mm in diameter without evidence of malignant change [6], and surgical excision for tumors >20 mm in diameter, benign GCTs causing symptoms, or when malignancy is suspected [18]. If no malignant changes are detected in the removed specimen, additional treatment or follow-up is not considered necessary [4].

EMR was recently reported to be an effective treatment in lesions similar in size to this case [19]. EUS was utilized to determine appropriateness for attempted endoscopic resection, as this technique is generally considered inappropriate for lesions greater than 2 cm in size or those with invasion into the muscularis propria. The treatment goal in this case was to attempt to endoscopically resect the area of dysplastic Barrett’s mucosa for definitive staging however it’s proximity to the GCT made resection of both lesions simultaneously the practical approach. Fortunately, this proved to be technically possible as both lesions were superficial to the muscularis propria.

The risk of regional metastatic disease with intramucosal cancer is on the order of 7-10% and this approaches the risk of major surgical morbidity and mortality in many centers [20,21]. Therefore EMR should be considered as an option in patients who are high risk for surgical resection, or who meet size criteria.

## Abbreviations

EMR: endoscopic mucosal resection
EUS: Endoscopic ultrasonography
GCT: Granular cell tumors
